# Anisomycin prevents OGD-induced necroptosis by regulating the E3 ligase CHIP

**DOI:** 10.1038/s41598-018-24414-y

**Published:** 2018-04-23

**Authors:** Mi-bo Tang, Yu-sheng Li, Shao-hua Li, Yuan Cheng, Shuo Zhang, Hai-yang Luo, Cheng-yuan Mao, Zheng-wei Hu, Jonathan C. Schisler, Chang-he Shi, Yu-ming Xu

**Affiliations:** 1 Department of Neurology, The First Affiliated Hospital of Zhengzhou University, Zhengzhou University, Zhengzhou, 450000 Henan China; 2 The Institute of Clinical Medicine, The First Affiliated Hospital of Zhengzhou University, Zhengzhou, China; 3 McAllister Heart Institute, Chapel Hill, NC 27514 USA; 4 Department of Cardiology, The University of North Carolina at Chapel Hill, Chapel Hill, NC 27514 USA

**Keywords:** Molecular biology, Cell death in the nervous system, Neurology

## Abstract

Necroptosis is an essential pathophysiological process in cerebral ischemia-related diseases. Therefore, targeting necroptosis may prevent cell death and provide a much-needed therapy. Ansiomycin is an inhibitor of protein synthesis which can also activate c-Jun N-terminal kinases. The present study demonstrated that anisomycin attenuated necroptosis by upregulating CHIP (carboxyl terminus of Hsc70-interacting protein) leading to the reduced levels of receptor-interacting protein kinase 1 (RIPK1) and receptor-interacting protein kinase 3 (RIPK3) proteins in two *in vitro* models of cerebral ischemia. Further exploration in this research revealed that losing neither the co-chaperone nor the ubiquitin E3 ligase function of CHIP could abolish its ability to reduce necroptosis. Collectively, this study identifies a novel means of preventing necroptosis in two *in vitro* models of cerebral ischemia injury through activating the expression of CHIP, and it may provide a potential target for the further study of the disease.

## Introduction

Necroptosis is a form of caspase-independent cell death, pathologically characterized by a gain in cell volume, swelling of organelles, plasma membrane rupture, and subsequent loss of intracellular contents^1–4^. Receptor-interacting protein kinase 1 (RIPK1), Receptor-interacting protein kinase 3 (RIPK3) and mixed lineage kinase domain-like (MLKL) play critical roles in the pathological activation of necroptosis^5,6^.

The type of cell death that ensues cerebral ischemia may include different programmed cell death mechanisms namely oxidative stress, ion balance disorder, calcium overload, apoptosis and necroptosis^7^. Studies have revealed that necroptosis contributed to the selective and delayed death of certain populations of neurons that occurs following an ischemic stroke^8^, which can be caused by a lack of blood supply to the brain^9^. Necroptosis has also been implicated in ischemic necrosis of retinal cells in several studies^10–12^. Oxygen-glucose deprivation (OGD), an *in vitro* model of cerebral ischemia, can also induce the death receptor (DR)-dependent component of necroptotic cell death in cultured neurons^13–15^, concomitant with the increase in RIPK3/RIPK1 mRNA and protein levels^16^. Similar phenomenons have been also detected in an *in vivo* model of transient global cerebral ischemia, specifically in the CA1 area^13^. These lines of evidence point to inhibiting necroptosis as a novel therapeutic strategy for cerebral ischemic injury, which represents the second leading cause of death as well as the leading cause of premature death and disability. Continued development of therapeutic options for ischemic stroke is woefully needed^17^.

C-terminus of HSC70-interacting protein (CHIP) is a 35-kDa protein that functions as both a molecular or autonomous chaperone and ubiquitin E3 ligase^18–20^. Previous studies have demonstrated that the TPR domain of CHIP underlined its cochaperone function, whereas the U-box domain of CHIP mediated its ubiquitin E3 ligase function^20^. The function of CHIP is proposed to behave as protective factor by degrading the abnormal folded proteins. Specifically, CHIP plays critical roles in the regulation of cell growth, apoptosis, neurodegeneration, invasion, stem cell differentiation, and cardiac fibrosis^21,22^. Study in the central nervous system indicated that overexpression of CHIP attenuated ER-stress death response while maintain ER stress adaptative response^23^. In brain ischemia, transfer of CHIP could prevent hippocampal neuronal death^24,25^. Recent studies have revealed that CHIP controls necroptosis through the ubiquitination and lysosome-dependent degradation of RIPK3 and RIPK1^26,27^. However, the role of CHIP in the necroptosis induced by OGD have not been described.

Ansiomycin is an inhibitor of protein synthesis which can also activate c-Jun N-terminal kinases. Study on macrophages have revealed that activation of JNK could induce the expression and activity of CHIP. While whether anisomycin could reduce OGD induced necroptosis have never been learned.

In the present study, whether necroptosis could be modified by anisomycin, a well-known activator of c-Jun N-terminal kinases(JNK)^28^, was first determined. Then a likely mechanism for anisomycin-mediated decreasing necroptosis via the upregulation CHIP in both OGD-challenged N2a cells and primary cultured hippocampal neurons was identified. Furthermore, whether CHIP regulated RIPK1 and RIPK3 degradation occurred in a co-chaperone and/or ubiquitin E3 ligase-dependent manner was assessed by using domain point mutants that disrupt the specific functions of CHIP in cellular model of OGD. The results of this study suggest that CHIP is a candidate therapeutic target for the treatment of necroptosis of ischemic stroke.

## Results

### OGD induced necroptosis in N2a cells

**Figure 1:**
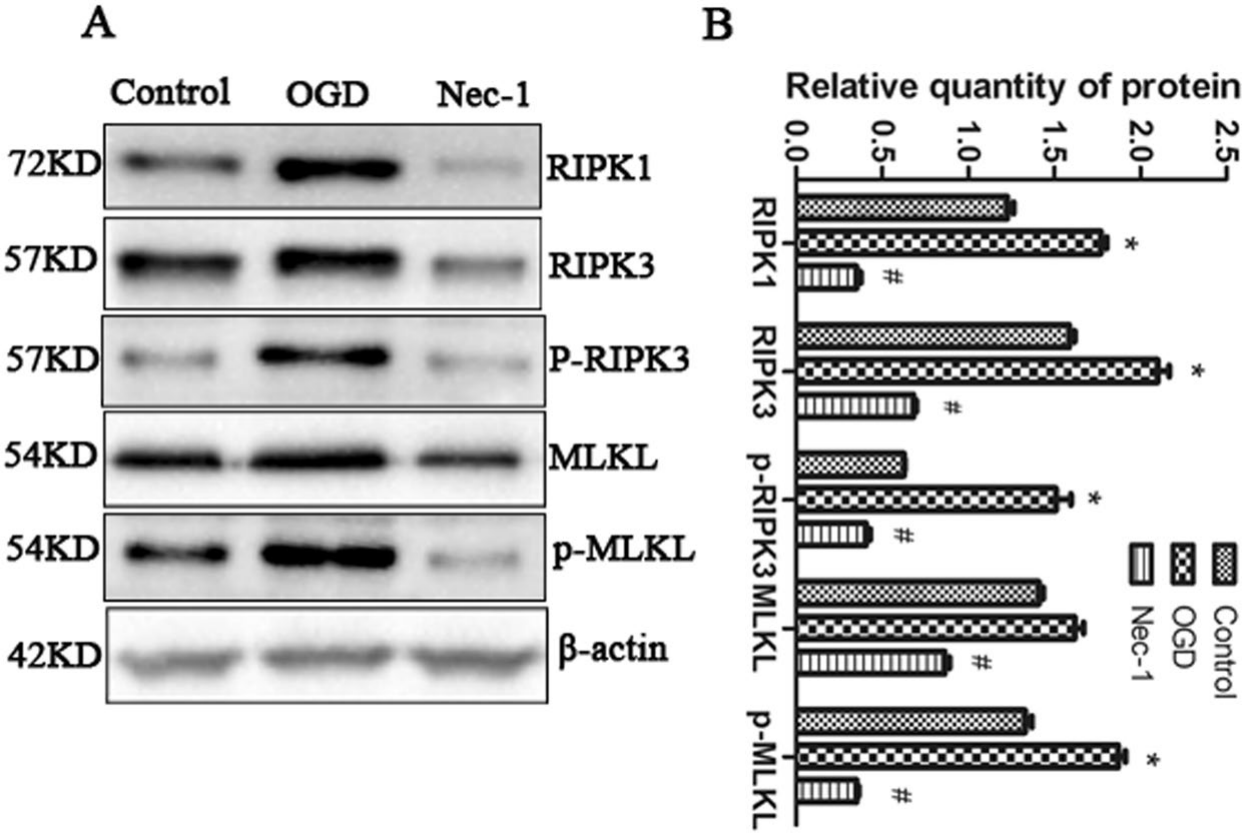
OGD challenge induces necroptotic cell death. (**A**–**B**) Representative western blot, with β-actin used for normalization. RIPK1, RIPK3, p-RIPK3 and p-MLKL in control cells, cells challenged by OGD which were pre-treated with or without Nec-1 (50 μM) were tested. The bars represent the mean ± SEM of five independent experiments. Significant differences *p < 0.05 vs. Control, ^#^*p* < 0.05 vs. OGD. Related blots are shown in Supplementary Fig. S1.

Studies indicated that necroptosis contributed to ischemic brain injury and neuronal death both *in vitro* and *in vivo*^13,29,30^. To establish cell-based model of necroptosis, N2a cells were challenged with oxygen-glucose-deprivation (OGD) 4 h with 12 h recovery with or without pretreatment with the RIPK1 inhibitor Nec-1. Immunoblot analysis demonstrated that OGD caused an increase in RIPK1, RIPK3, p-RIPK3 and p-MLKL (Fig. 1A,B). Furthermore, this increase in RIPK1, RIPK3, p-RIPK3 and p-MLKL were abrogated by pretreatment with Nec-1 (Fig. 1A,B), suggesting that OGD challenging in a neuronal cell line activates the necroptosis. The data revealed that targeting pathways that can blunt or inhibit necroptosis induced by OGD may prevent additional cell death.

### Anisomycin reduced OGD-induced necroptosis in N2a cells

**Figure 2:**
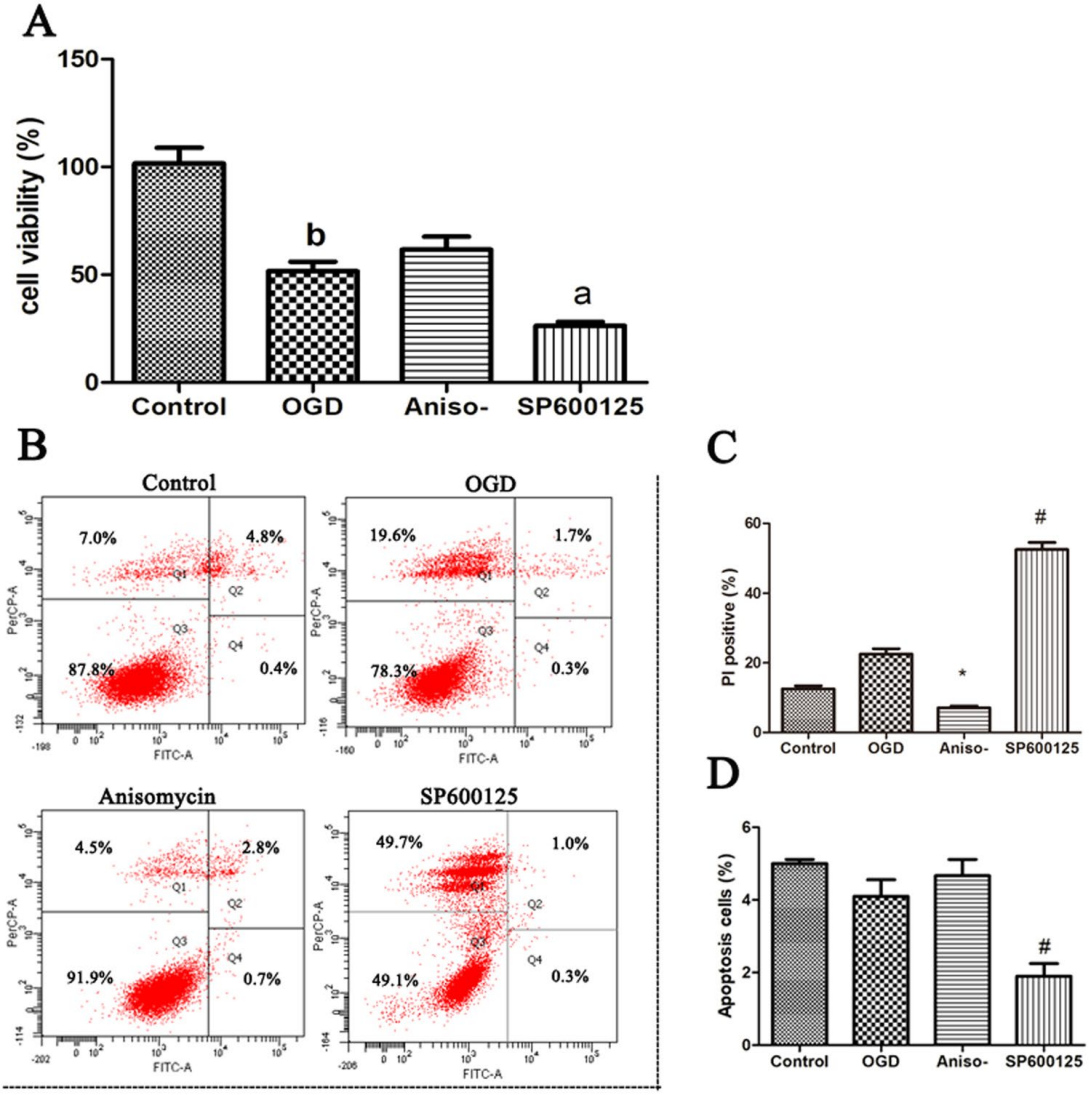
Anisomycin reduces necroptosis of N2a cells following OGD. 24 h prior to an OGD challenge, N2a cells were treated with or without 100 nM anisomycin or 10 μM SP600125. After 4 h OGD followed with 12 h recovery, tests were implemented ** (A)** Cell counting kit was used to test the viability of each group. Significant differences ^b^*p* < 0.05 vs. Control, ^a^*p* < 0.05 vs. OGD **(B)** AnnexinV-FITC Apoptosis Detection Kit was used to test the necroptosis and apoptosis cells. **(C)** Propidium iodide (PI)-positive cells were analyzed after a 12 h recovery of OGD. Bars represent the mean ± SEM of four independent experiments. Significant differences *p < 0.05 vs. OGD, ^#^*p* < 0.05 vs. OGD. **(D)** Apoptosis cells were analyzed after 4 h OGD and 12 h recovery. Bars represent the mean ± SEM of four independent experiments. Significant differences ^#^*p* < 0.05 vs. OGD.

There are conflicting reports on the role of JNK in cell death^31,32^, suggesting the role of JNK may be stress on cell-type dependent. To test the effect of JNK in OGD model, N2a cells were pre-treated with either the JNK agonist, anisomycin, or the JNK inhibitor, SP600125, followed by OGD-challenge. Cell viability was tested after OGDR. The result showed that OGDR decreased the cell viability compared with control cells. While pre-treatment anisomycin rescued the viability of OGDR cells, though with non-significance. Pre-treatment SP600125 aggravated the injury of OGD (Fig. 2A). Further, apoptosis and necroptosis were also tested. Results showed that anisomycin inhibited OGD-induced necroptosis as evidenced by a reduction in the ratio of PI-positive N2a cells when compared to OGD only treated cells or cells pretreatment with SP600125 before OGD. Cells pre-treatment SP600125 aggravated the necroptosis compared with the OGD only treatment cells (Fig. 2B,C). Simultaneous, lower apoptotic cells after OGD treatment has also been detected compared with cells cultured in normal condition, though with non-significance. Anisomycin pretreatment cells to some extent increased apoptosis cells, though with non-significance. SP600125 pretreatment cells expectedly reduced apoptosis cells (Fig. 2D). Thus, the mechanism by which anisomycin protects cells from OGD-induced necroptosis represented a possible therapeutic and study pathway to interrogate.

### Anisomycin treatment increased CHIP expression while reducing RIPK3 expression in OGD-challenged N2a cells and primary hippocampal neurons

**Figure 3:**
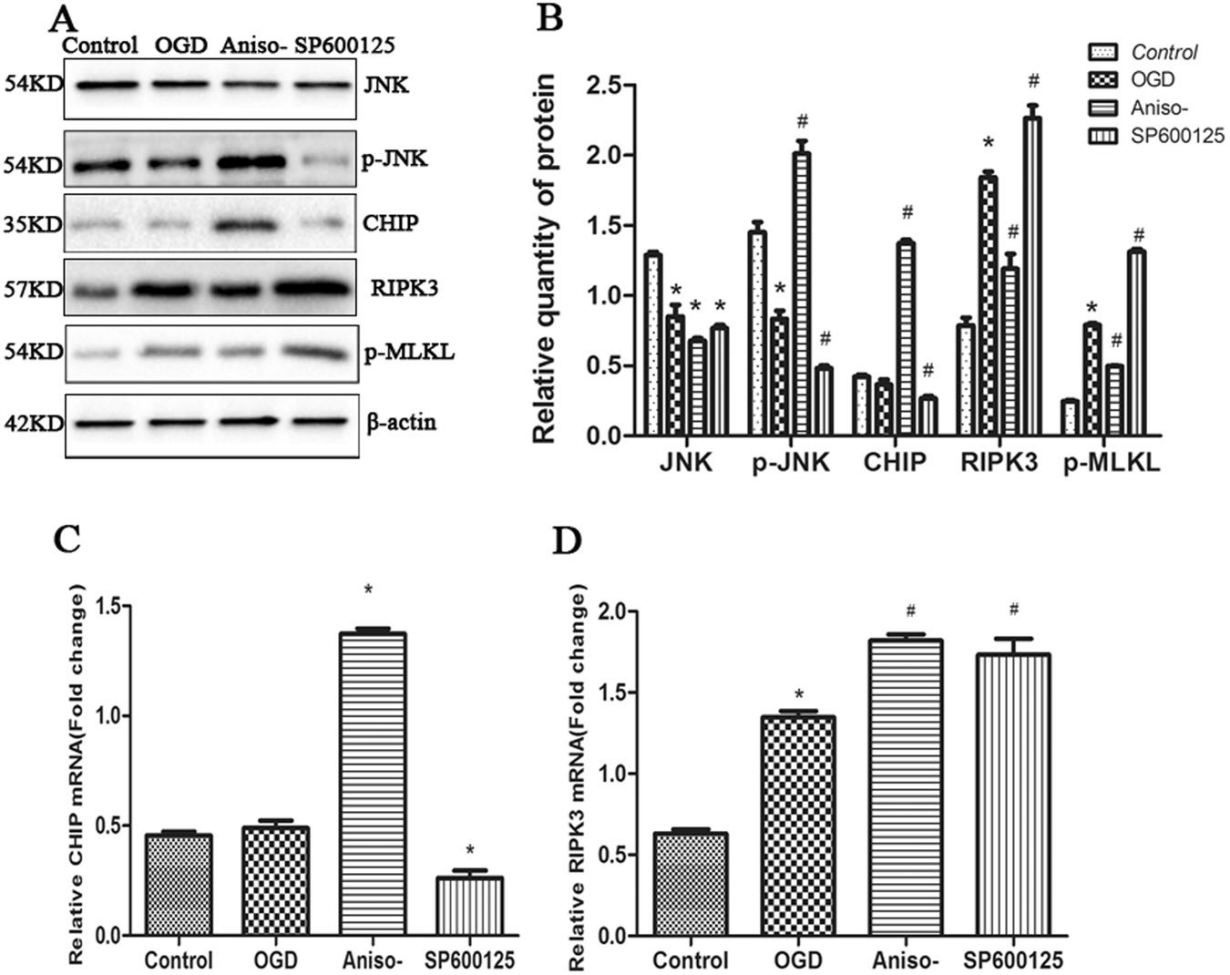
Anisomycin treatment increased CHIP expression while reducing necroptosis in OGD-challenged N2a cells. **(A)** Cells managed as Fig. 2. 12 h recovery after OGD, the relative protein levels of total JNK, phospho-JNK, RIPK3, p-MLKL and CHIP were measured by western blotting (left-hand panel, representative blot). **(B)** Semi-quantitative analysed of relative proteins (right-hand panel, bars represent the mean ± SEM of eight independent experiments). Significant differences *p < 0.05 vs. Control, ^#^*p* < 0.05 vs. OGD. **(C**) 24 h prior to an OGD challenge, N2a cells were treated with or without 100 nM anisomycin, or 10 μM SP600125. After a 12 h recovery, q-RT-PCR was used to quantitate the relative mRNA levels of *CHIP*. The bars represent the mean ± SEM of five independent experiments. Significant differences *p < 0.05 vs. OGD. **(D**) 24 h prior to an OGD challenge, N2a cells were treated with or without 100 nM anisomycin, or 10 μM SP600125. After a 12 h recovery of OGD, q-RT-PCR was used to quantitate the relative mRNA levels of *RIPK3*. The bars represent the mean ± SEM of five independent experiments. Significant differences *p < 0.05 vs. Control, ^#^*p* < 0.05 vs. OGD. Related blots are shown in Supplementary Fig. S2.

**Figure 4:**
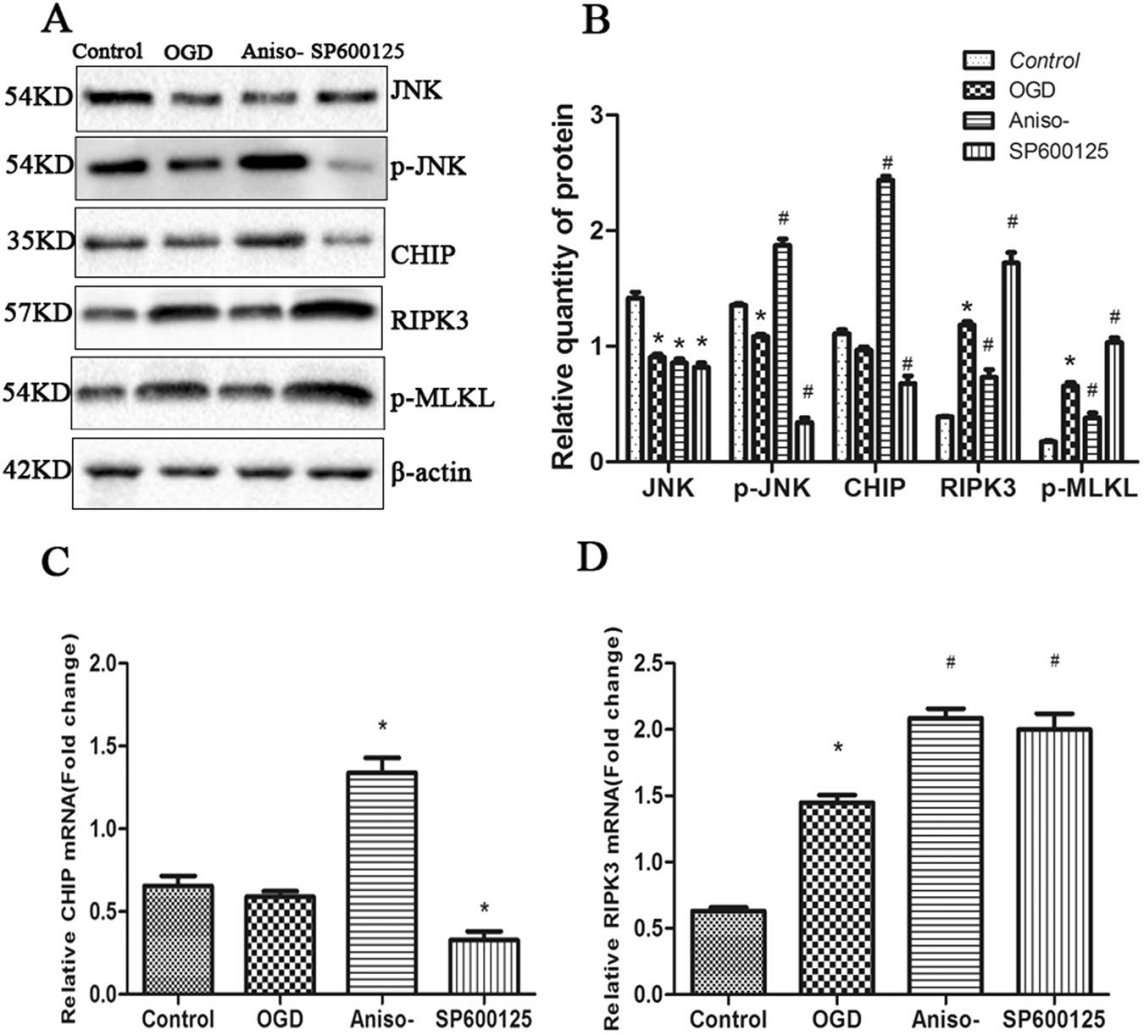
Anisomycin upregulates CHIP and functions as an anti-necroptotic factor in OGD-challenged primary cultured hippocampal neurons. Primary cultured hippocampal neurons were treated with or without 50 nM anisomycin, or 10 μM SP600125 at the 7th day of culture *in vitro*. (**A**) After 4 h OGD insulted and recovery for 12 h, lysates from the above cells were harvested. The indicated proteins were detected by Western blot. The protein levels of total JNK, phospho-JNK, RIPK3, p-MLKL and CHIP were measured by western blotting (left-hand panel, representative blot) and semi-quantitative analysed (right-hand panel, bars represent the mean ± SEM of eight independent experiments). (**B**) Semi-quantitative analysed (right-hand panel, bars represent the mean ± SEM of eight independent experiments). Significant differences *p < 0.05 vs. Control, ^#^*p* < 0.05 vs. OGD. (**C**) 24 h prior to an OGD challenge, cells were treated with or without 50 nM anisomycin, or 10 μM SP600125. After a 12 h recovery, q-RT-PCR was used to quantitate the relative mRNA levels of *CHIP*. The bars represent the mean ± SEM of five independent experiments. Significant differences *p < 0.05 vs. OGD. (**D**) 24 h prior to an OGD challenge, cells were treated with or without 50 nM anisomycin, or 10 μM SP600125. After a 12 h recovery, q-RT-PCR was used to quantitate the relative mRNA levels of *RIPK3*. The bars represent the mean ± SEM of five independent experiments. Significant differences *p < 0.05 vs. Control, ^#^*p* < 0.05 vs. OGD. Related blots are shown in Supplementary Fig. S3.

A previous study implicated a direct role for protein quality control and in attenuating necroptosis through the ubiquitin and lysosome-dependent degradation of RIPK3^26^. However, to date, the role of CHIP in OGD-challenged cells has not been explored. As expected, a reduction of JNK in OGD challenged cells was observed compared to un-challenged N2a cells. As expected, pre-treatment cells with 100 nM anisomycin before OGD increased the p-JNK compared to OGD treated only cells (Fig. 3A,B). Furthermore, immunoblot analysis of lysates from anisomycin pre-treatment cells revealed an increase of CHIP protein compared with OGD treated only cells, contrasting with a decrease in RIPK3 and p-MLKL protein (Fig. 3A,B). Reciprocally, pre-treatment cells with SP600125 resulted in the expected attenuation in JNK phosphorylation levels, as well as decreased CHIP protein levels and rising RIPK3, p-MLKL (Fig. 3A,B). To determine if the changes in steady-state protein levels were caused by transcription, the relative mRNA levels of CHIP and RIPK3 were tested by quantitive PCR (q-PCR). The changes in CHIP mRNA seen with JNK activation and inhibition reflected what was observed at the protein level (Fig. 3A,C), suggesting that JNK signaling may regulate CHIP transcription. As expected, a robust increase in RIPK3 transcription in response to SP6001125 was measured, which coincided with an increase in RIPK3 protein (Fig. 3A,D). Remarkably, despite the protective effects of anisomycin on preventing OGD-induced necroptosis and the reduction observed in RIPK3 protein (Fig. 3A), anisomysin treatment increased RIPK3 mRNA compared to OGD insulted only cells (Fig. 3D), suggesting that the decrease of RIPK3 protein and attenuation of cell necroptosis via anisomysin is likely due to the post-translational regulation of RIPK3. Furthermore, these data suggest that the responsiveness of CHIP regulation to JNK signaling during OGD may mediate the degradation of RIPK3. We subsequently extended the results in an OGD model using primary neurons isolated from the rat hippocampus and observed similar results to the N2a cells regarding the effect on CHIP, RIPK3 and p-MLKL when targeting the JNK pathway (Fig. 4)

### Anisomycin increase the endogenous RIPK3 ubiquitination level in OGD challenged N2a cells

**Figure 5:**
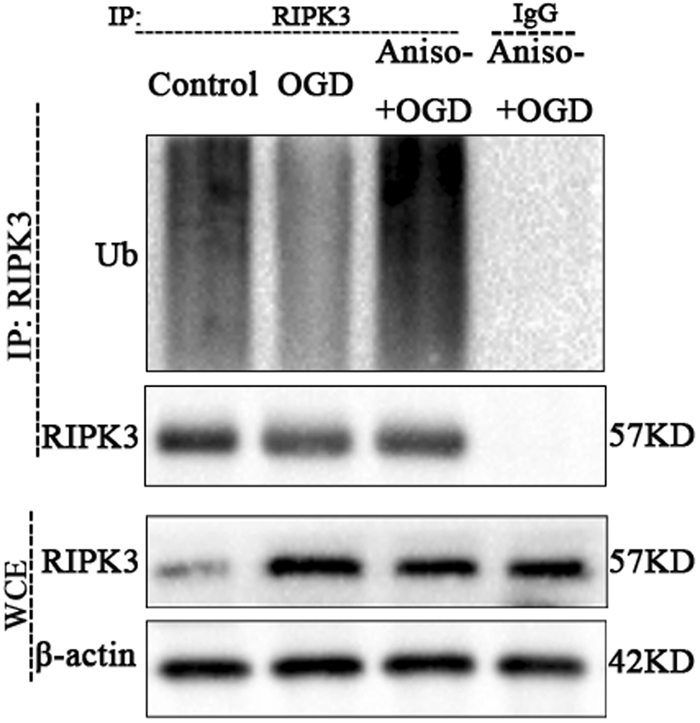
Anisomycin increase the endogenous RIPK3 ubiquitination level in OGD challenged N2a cells. Control cells, OGDR insulting cells and OGD challenged with anisomycin pre-treatment cells were cultured. They were incubated for the last 6 h with the proteasome inhibitor MG132 (10 μM). RIPK3 were immunoprecipitated with anti-RIPK3 and IgG antibodies for ubiquitination analysis. Related blots are shown in Supplementary Fig. S4.

The opposing changes in the levels of RIPK3 mRNA and protein in anisomycin pre-treatment and OGD insulted N2a cells promoted us to tested the ubiquitination and degradation of RIPK3. Control cells, OGD insulted cells and anisomycin pre-treatment OGD insulted cells were harvested for detecting the endogenous RIPK3 ubiquitination levels. The result showed that OGD challenged cells have a lower level of endogenous RIPK3 ubiquitination compared with control (Fig. 5). Anisomycin could increase the level of endogenous RIPK3 ubiquitination compared to OGD insulted cells (Fig. 5). The data revealed that anisomycin could reduce the stability of RIPK3 by ubiquitination.

### CHIP functions as an anti-necroptotic factor in OGD-challenged cells by post-translational regulation of RIPK3

**Figure 6:**
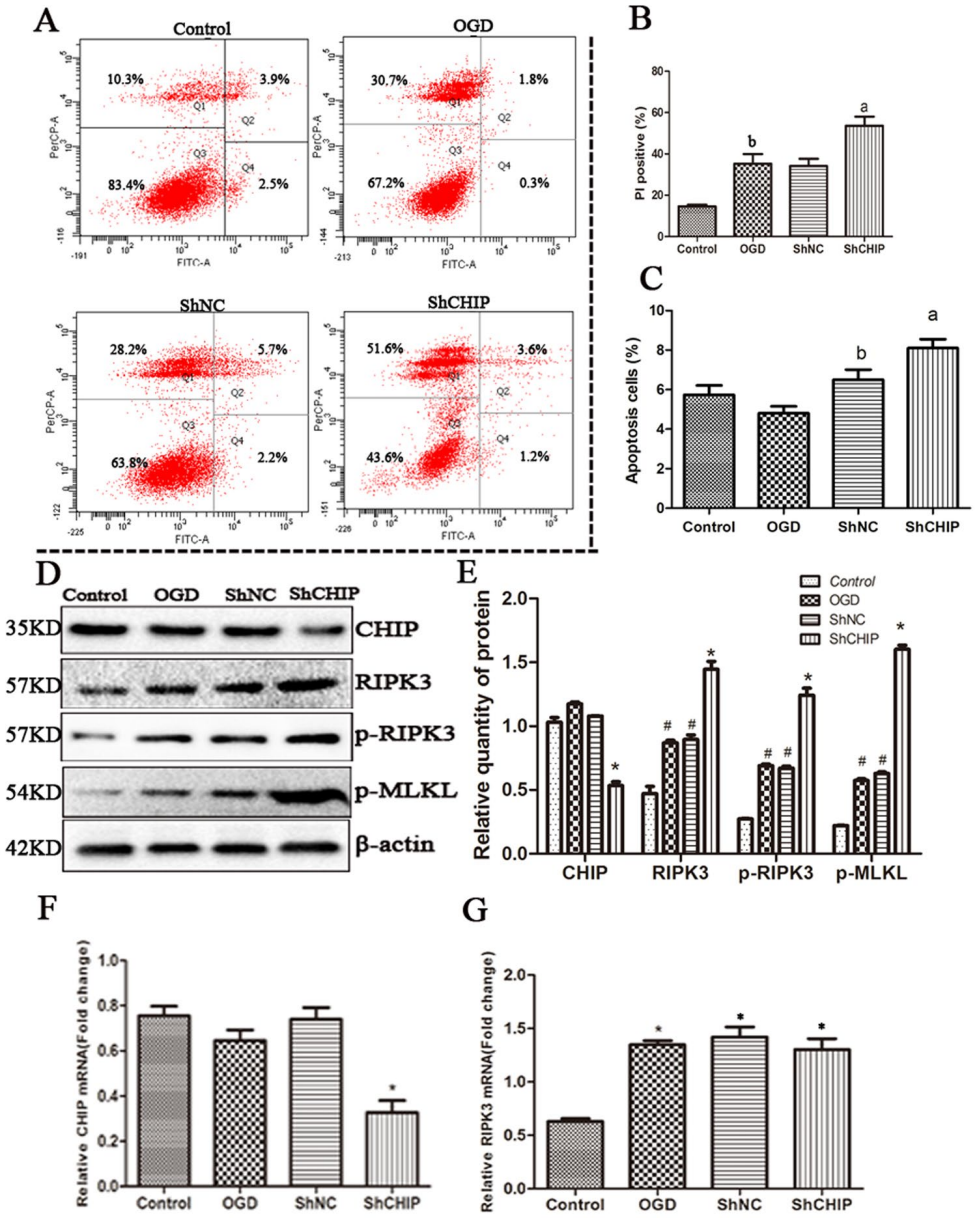
CHIP functions as an anti-necroptotic factor in OGD-challenged cells **(A)** 24 h after transfection of N2a cells with PGPU6-GFP-shNC or PGPU6-GFP-si-CHIP, cells were challenged with OGD for 4 h. After 12 h of recovery, the population of PI positive cells and apoptosis cells were determined using flow cytometry using the AnnexinV-FITC Apoptosis Detection Kit. **(B)** The population of PI positive cells. Bars represent the mean ± SEM of nine independent experiments. Significant differences ^b^*p* < 0.05 vs. Control, ^a^*p* < 0.05 vs. shNC. **(C)** The population of apoptosis cells. Bars represent the mean ± SEM of nine independent experiments. Significant differences ^a^*p* < 0.05 vs. ShNC, ^b^*p* < 0.05 vs. OGD **(D**,**E)** Representative western blot assessing of CHIP, RIPK3, p-RIPK3, p-MLKL in transfected cells were tested after transfection and OGD challenging. Bars represent the mean ± SEM of nine independent experiments. Significant differences *p < 0.05 vs. ShNC, ^#^*p* < 0.05 vs. Control. **(F)** q-RT-PCR analysis of *CHIP* mRNA levels in transfected and OGD insulted cells. *p < 0.05 vs. ShNC. **(G)** q-RT-PCR analysis of *RIPK3* mRNA levels in transfected and OGD insulted cells. *p < 0.05 vs. Control. Related blots are shown in Supplementary Fig. S5.

The opposing changes in the levels of CHIP and RIPK3 are consistent with a previous study identifying RIPK3 as an ubiquitinated substrate of CHIP^27^. However, to determine if the increase in CHIP expression observed with anisomycin treatment is necessary to reduce necroptosis in OGD insulted cells model, short interfering RNA was used to reduce CHIP expression in OGD insulted N2a cells. Consistent with a protective role for CHIP in OGD, the number of cells undergoing necroptosis almost doubled when transfected with ShCHIP compared to cells transfected with a mock plasmid, ShRNA (ShNC) (Fig. 6A,B). Simultaneous, an increase of apoptosis cells has also been seen in CHIP knock down cells compared with ShNC cells (Fig. 6C). The 70% decrease in CHIP protein levels seen in cells transfected with ShCHIP (Fig. 6D,E) was accompanied by increase in RIPK3 and p-RIPK3 at the protein but not transcript level when compared with ShNC cells (Fig. 6D–G), consistent with our previous analyses (Figs 3 and 4). The above data suggest that CHIP may functions as an anti-necroptotic factor and could inhibit necroptosis following OGD challenge.

### The important role of CHIP in reducing RIPK3 in anisomycin treatment OGD-challenged N2a cells

**Figure 7:**
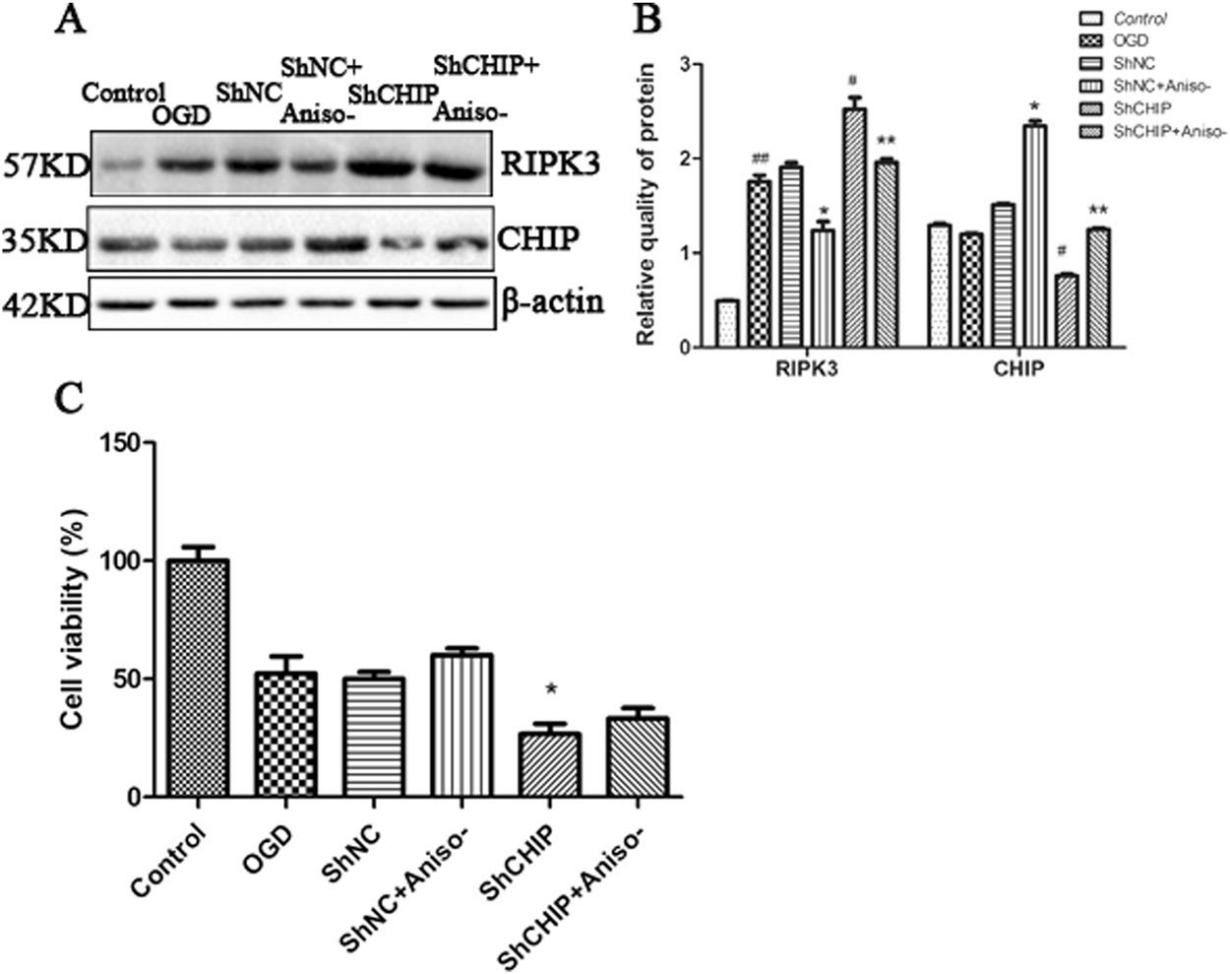
CHIP is important for reducing RIPK3 levels in anisomycin treated OGD-challenged N2a cells. 24 h after transfection of N2a cells with PGPU6-GFP-shNC or PGPU6-GFP-siCHIP, cells were challenged with OGD for 4 h after treat with or without anisomycin for 24 h. **(A)** With 12 h of recovery, lysates of OGDR insulted N2a cells were analyzed by western blot to detect the quantitation of RIPK3. **(B)** The lysates from (A) were respectively loaded in the gels and analyzed by western blot to detect the quantitation of CHIP and RIPK3. Bars below the blots represent the mean ± SEM of four independent experiments. Significant differences ^##^*p* < 0.05 vs. Control, *p < 0.05 vs. ShNC, ^#^*p* < 0.05 vs. ShNC, **p < 0.05 vs. ShCHIP. Related blots are shown in Supplementary Fig. S6. **(C)** Cell viability was tested after different treatment. Significant differences *p < 0.05 vs. ShNC.

As anisomycin is also an inhibitor of protein synthesis, and there was a study showed that inhibiting protein synthesis was protective for cells, thus the role of protein synthesis inhibition should be taken into account. As expected, pre-treatment anisomycin reduced RIPK3 in shNC transfected cells. While in shCHIP transfected cells, pre-treatment anisomycin only partly reduced RIPK3 (Fig. 7A,B). The cell viability showed that knock down CHIP deteriorated the OGD injury compared to ShNC, and anisomycin treatment could still decrease the injury of OGD at some extent (Fig. 7C), though with non-significance. The above results illustrated that other function of anisomycin, may be inhibition of protein synthesis, benefit to the OGD induced necroptosis. While the role of CHIP in anisomycin reducing necroptosis is much more important.

### CHIP as an anti-necroptotic factor requires both co-chaperone and ubiquitin ligase functions

**Figure 8:**
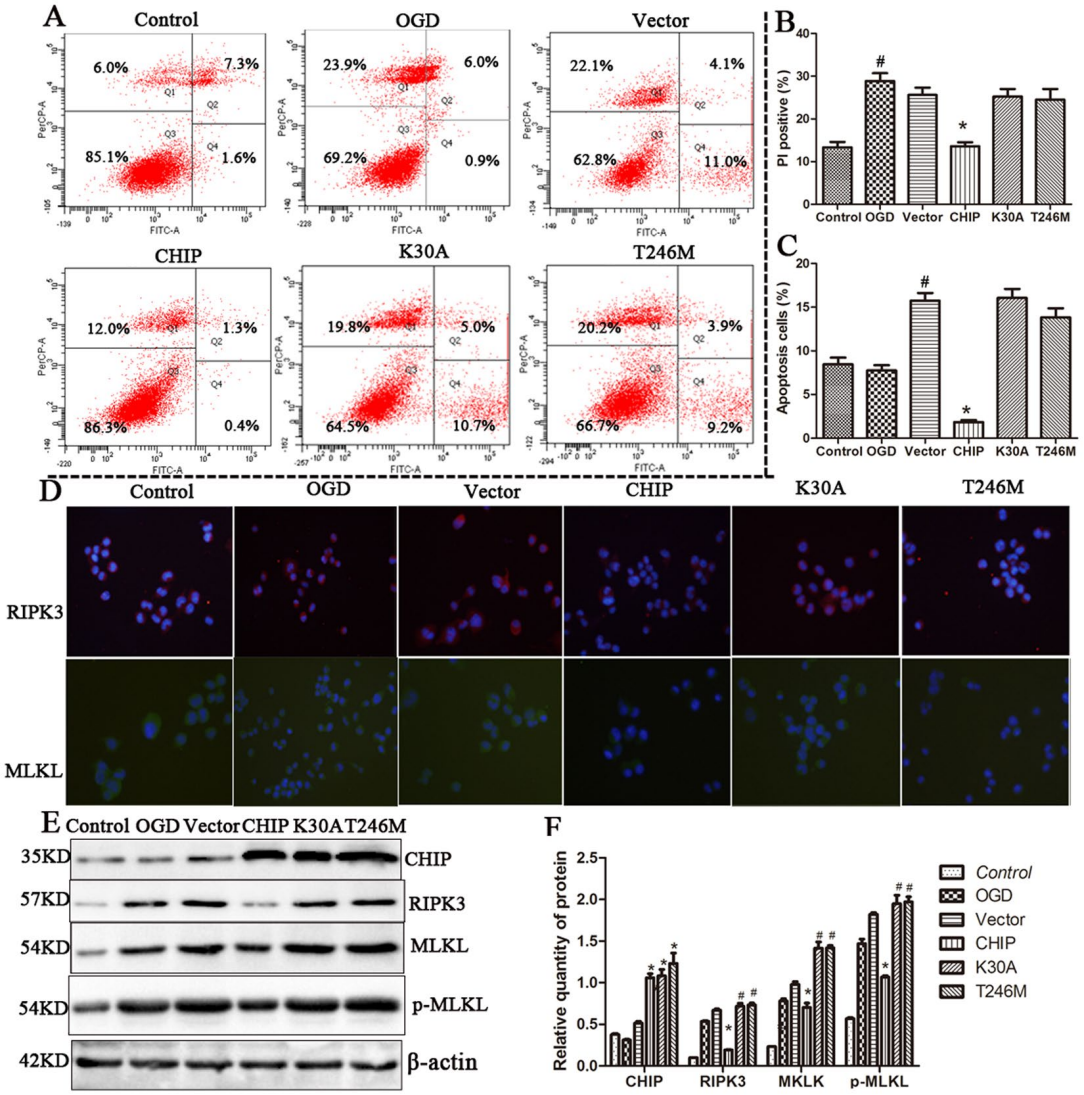
CHIP functions as an anti-necroptotic factor in a domain-dependent manner **(A)** N2a Cells transfected with wild-type CHIP and different domain mutants of CHIP (K30A, T246M) were analyzed for ratio of PI positive cells and apoptosis cells by Flow cytometry after OGDR **(B)** PI positive cells were analyzed. Bars represent the mean ± SEM of four independent experiments. Significant differences ^#^*p* < 0.05 vs. Control, *p < 0.05 vs. Vector. **(C)** Apoptosis cells were analyzed. Bars represent the mean ± SEM of four independent experiments. Significant differences ^#^*p* < 0.05 vs. OGD, *p < 0.05 vs. Vector. **(D)** Immunofluorescence showing expression of RIPK3 (red) and MLKL (green) in cells transfected as above. Nuclei are stained in blue. Scale bar, 10 μm. **(E**,**F)** Representative western blot (left hand panel) of cells transfected with *CHIP* and *CHIP* mutants showing the expression of CHIP, RIPK3, MLKL and p-MLKL. The right-hand panel showed a semi-quantitative analysis using β-actin for normalization (bars represent the mean ± SEM of four independent experiments). *p < 0.05 vs. Vector, ^#^*p *< 0.05 vs. CHIP. Related blots are shown in Supplementary Fig. S7.

The data directly implicate CHIP as a protective factor in OGD. The multi-functional properties of CHIP in protein quality control prompted us to further study the molecular mechanism underlying CHIP function during OGD. In particular, the discordance observed between the mRNA and protein levels of RIPK3 with increased CHIP expression suggested that post-transcriptional mechanisms and protein degradation may play important roles. To assess the distinct functions of CHIP in this process, mutations that caused loss of function in either the TPR or U-box domains of CHIP were constructed and used to transfect N2a cells which were subjected to OGD challenge. Cells expressing exogenous wild-type CHIP had a 40% decrease in the number of PI-positive and 50% decrease in the number of apoptosis compared to cells transfected with an empty vector, whereas the exogenous expression of the other CHIP variants had little to no protection on necroptosis (Fig. 8A–C). Immunoblot analysis of cell lysates and indirect immunofluorescence from these same conditions demonstrated that only wild-type CHIP affected the steady-state protein levels of RIPK3 and p-MLKL (Fig. 8D–F). Loss of either the co-chaperone function of CHIP (K30A) or ubiquitin ligase function (T246M) ablated the protective function of CHIP on cell necroptosis (Fig. 8A,E). These data suggest that both the co-chaperone and ubiquitin E3 ligase functions of CHIP are essential for anti-necroptotic in a cellar model of cerebral ischemia.

## Discussion

Cerebral ischemia is the leading neurological cause of death and long-term disability worldwide^33,34^. With numerous pathological mechanisms implicated in the ultimate outcome of ischemic stroke, one of these mechanisms is necroptosis, which may reveal a potential novel target for generating new therapeutic strategies for this disease^13^. To date, studies that focus on inhibiting necroptosis in the context of cerebral ischemia are limited.

As a novel type of programmed cell death, necroptosis continues to be extensively studied and implicated in a wide range of pathologies, especially in neurodegeneration disease, such as Parkinson’s disease^35,36^, Huntington’s Chorea and Niemann–Pick disease^3,36^. Recently, necroptosis has also been revealed to mediate the process of delayed ischemic brain injury^8,13,37^. Some suggests that necroptosis mediate oligodendrocytes death in the developing brain^38^. Others hold that RIPK3 induces ischemic neuronal DNA degradation and programmed necrosis in rat via apoptosis-inducing factor^39^. However, the related pathogenic mechanism of the regulation of necroptosis in cerebral ischemia has not been comprehensively explored. Using *in vitro* models of cerebral ischemia, the present study demonstrated that anisomycin, a JNK activator, can protect neuronal cells from necroptosis largely by increasing the expression of E3 ligase CHIP. Further, the ability of CHIP to reduce the level of necroptosis dependeds on both its cochaperone and ubiquitin E3 ligase function. These results elucidated a novel mechanism of protection of necroptosis in cerebral ischemia and may pave a road towards new therapies to treat cerebral ischemia.

Anisomycin is an activator of JNK, a member of the mitogen activated protein kinase (MAPK) superfamily^40^.SuperscriptOf the three family members, JNK3 is the isoform expressed in cardiac and neuronal tissues. While the role of JNK and the molecular mechanisms involved in the process of necroptosis have never been fully explored, especially under conditions of OGD. In the present study, using an *in vitro* model of cerebral ischemia, anisomycin prevented the OGD-stimulated necroptosis, as evidenced by the much lower ratio of PI-positive cells compared to OGD challenged only cells. In previous studies on macrophages have concluded that it was through activation of JNK, that Toll-like receptor 2 (TLR2) enhanced the expression and activity of CHIP^41^. In the present cell-based models of cerebral ischemia study, the regulation of CHIP in a JNK-dependent manner was also observed. Anisomycin treatment increasing the expression of CHIP in the present study is consistent with the previous study^41^. Even though it has been revealed that anisomycin inhibit protein synthesis by activating phosphorylation of eukaryotic elongation factor 2 (eEF2)^42^, which  may contribute to the protection, recent study in the central nervous system has shown that overexpression of CHIP could abrogated eIF2-α phosphorylation in condition of endoplasmic reticulum stress^23^. Thus, we speculated that the JNK upregulating CHIP may abrogate eIF2-α phosphorylation and further, to some extent, impaired its foundation of protein synthesis inhibition. The present result revealed that the protection function may mostly belong to the upregulation of CHIP. Simultaneously, there are complicated cross-talk among different signal pathways and different condition. Study in the case of non-alcoholic steatohepatitis demonstrated that JNK inhibitor suppresses the expression of RIPK3^43^. While in the present study, JNK activation reduced the level and activation of RIPK3 by upregulating CHIP. The event may be attribute to that JNK could activate many transcription factors like c-Jun, ATF2, Elk, p53, c-Myc, and these transcriptions may have potential to regulate the expression of CHIP. On the other hand, the direct interaction between JNK and the promoter of CHIP also need to be verified. All in all, the negative association between CHIP and RIPK3 observed in the present study are consistent with previous studies that demonstrated that the degradation of RIPK3 protein is regulated via CHIP^26^. The study further demonstrated that both the cochaperone and ubiquitin E3 ligase functions were essential for the degradation of RIPK3. Though controversies exist over the role of JNK in cell death, primarily whether it has a pro-apoptotic or an anti-apoptotic role, in the present study, anisomycin induced activation JNK increased apoptosis, though without obvious statistical significance. Though the increase of viability of anisomycin-treatment was non-significant, it provides a novel potential target for the further study of ischemic stroke.

In conclusion, a potential target for the further study and prevention of ischemic stroke has been identified in the present study. Anisomycin, an activator of JNK, can block the necroptotic pathway by upregulating the expression of CHIP under OGD conditions. Continued understanding of the role of JNK in CHIP-mediated necroptosis will provide further insight and may contribute to the development of drugs related to necroptosis-associated ischemic stroke.

## Materials and Methods

### Animals

The 18-day pregnant Sprague-Dawley rats were provided by the Animal Experimental Center of Zhengzhou University. All the experiments were performed in accordance with animal welfare guidelines at the Zhengzhou University and were approved by the institutional animal care and use committee.

### Materials

The antibodies against RIPK1 (SAB3500420), RIPK3 (Sc-374639) and RIPK3 (ab56164) were purchased from Sigma-Aldrich (St. Louis, MO, USA), Santa Cruz Biotechnology (Santa Cruz, CA, USA) and Abcam (Cambridge, MA, USA), respectively. The antibody against β-actin (T4026) was from Sigma-Aldrich, the antibody JNK (mAb#4668) was from Cell Signaling Technology (CST, Beverly, MA, USA), The antibody against CHIP (55430-1-AP) was from Proteintech (WuHan, China), anisomycin (HY-18982) and the JNK inhibitor SP600125 (HY-12041) were from MCE (Monmouth Junction, NJ, USA) and AnnexinV-FITC Apoptosis Detection Kit was from Vazyme (Nanjing, China). The wild-type CHIP (pcDNA3.1-CHIP) was obtained from Addgene (Cambridge, MA, USA), the vector pcDNA3.1, mutants of CHIP in pcDNA-3.1-K30A/T246M, PGPU6-GFP -shNC, and PGPU6-GFP-sh-CHIP were all constructed by GenePharma (Shanghai, China).

### Cells culture and transfection

The cell line N2a was obtained from the China Infrastructure of Cell Line Resources and cultured in Dulbecco’s modified Eagle’s medium (Invitrogen, Waltham, MA, USA) containing 10% heat-inactivated fetal bovine serum (Gibaco, Gaithersburg, MD, USA), 100 U/mLpenicillin, and 100 μg/mLstreptomycin (Hyclone, Logan, UT, USA) under 5% CO_2_ at 37 °C.

Primary cultures of hippocampal neurons were prepared as previously described. The cells were taken from 18-day pregnant Sprague-Dawley rats, as previously described^44^. Briefly, the flake-shape hippocampi were dissociated with trypsin in Hank’s Balanced Salt Solution. Neurons were cultured in 24-well plates coated with poly-D-lysine, in Neurobasal® medium (Life Technologies™, Carlsbad, CA, USA) supplemented with 2% NeuroCult™ SM1 Neuronal Supplement (StemCell™ Technologies, Grenoble, France), 0.5 mM glutamine, 0.125 μg/mL gentamicin, and 25 μM glutamate. Cells were maintained at 37 °C in a humidified incubator with 5% CO_2_ for up to 7 days *in vitro*. On the 7th day, the neurons were fed with fresh supplemented Neurobasal medium without glutamate and treated with anisomycin and SP600125 24 h prior to OGD challenge.

According to the manufacturer’s instructions, equal quantities of the plasmids (vector, CHIP, and the CHIP mutants K30A, T246M) were each transfected into N2a cells using Lipofectamine^TM^ 2000 (Invitrogen, USA). Cells transfected with the pcDNA3.1 vector were cultured as a control.

### Oxygen-glucose deprivation (OGD) challenge

After transfection and culture for 24 h, cells were placed in a glucose-free-deoxygenated buffer medium (OGD medium: 10 mM HEPES, 116 mMNaCl, 5.4 mMKCl, 0.8 mM NaH_2_PO_4_, 25 mM sodium bicarbonate, 25 mM sucrose, 1.8 mM CaCl_2_, and 0.04% phenol red, pH 7.3) inside an anaerobic chamber with 5% CO_2_ and 95% N_2_ (OGD chamber, Thermo Forma 1029, Thermo Fisher Scientific, Waltham, MA, USA), at 37 °C. Thus, there was no oxygen in this environment. N2a cells were OGD challenged for 4 h and primary cultures of hippocampal neurons were OGD challenged for  4h. After incubation, the cells were placed in their conditioned medium and returned to the normoxic incubator for a period of recovery lasting up to 12 h. In the OGD necroptotic cell model, 100 nM anisomycin and 10 μM SP600125 for N2a cells, and 50 nM anisomycin and 10 μM SP600125 for primary hippocampal neurons (the volume of agent is less than 1/1000), were added to the medium 6 h prior to OGD and were present during both the stimulation and post-stimulation periods at the indicated concentrations.

### Cell viability assay

Cells were reseeded into 96-well plates and plated in triplicate wells for all conditions. Before tested the cell viability, 10 μl of Cell Counting Kit 8 (DojinDo, Japan) was added to each well. Mixtures were incubated for 2 h. Absorbance at 450 nm was measured using a BioTek Synergy H1 Hybrid Multi-Mode Microplate Reader (BioTek, Vermont, USA)

### RNA extraction and quantitative real time reverse transcription-polymerase chain reaction (q-RT-PCR) analysis

Total RNA from the OGD-induced necroptosis cell model was extracted using TRIzol® Reagent (Invitrogen), according to the manufacturer’s specifications. The total amount of RNA was quantified spectrophotometrically (Thermo Nanodrop 2000, Wilmington, DE, USA), and the RNA integrity and quality were evaluated using the Experion automated gel electrophoresis system (Bio-Rad Laboratories, Berkeley, CA, USA).

For cDNA synthesis, 500 ng of total RNA was used with the HiScript® II One Step q-RT-PCR SYBR® Green Kit (Vazyme Biotech, Nanjing, China), according to the manufacturer’s instructions. For quantitative real-time PCR (q-RT-PCR), 20 μL reactions were prepared with 2 μL of 1:10 diluted cDNA, 10 μL of AceQqPCR SYBR Green Master Mix (Vazyme, China) and specific primers at 100 nM, as follows: β-actin forward–5′-TACAGCTTCACCACCACAGC-3′, reverse–5′-TCTCCAGGGAGGAAGAGGAT-3′; RIPK3 forward–5′-GGGACCTCAAGCCCTCTAAC -3′, reverse–5′-GATCCTGATCCTGACCCTGA-3′; CHIP forward–5′-AAGAGCTCAAGGAGCAGGGAA-3′ reverse–5′-TGTTCAGGCTGCTGCATCTTC-3′ for N2a cells; β-actin forward–5′- TACAGCTTCACCACCACAGC-3′, reverse –5′-TCTCCAGGGAGGAAGAGGAT -3′; RIPK3 forward–5′- GGAGGTGAAGGCTATGGTGA -3′, reverse–5′- CCGTTCTCCATGAATCCTGT-3′; CHIP forward–5′- AAGAAGAAGCGCTGGAACAG-3′ reverse–5′- GTGGTTCCGCTGACACTCTT-3′ for hippocampal neurons. The fluorescent signal was measured after each elongation step of the PCR reaction in the StepOne^TM^ and StepOnePlus^TM^ Real-time PCR Systems (Thermo Fisher, USA), and used to determine the threshold cycle (Ct) as previously described^45^. All melting curves were performed to detect non-specific amplification products. In all assays, non-template controls were included, and for each set of primers, a standard curve was constructed to assess primer efficiency. Reactions were run in quadruplicate. The level of each gene was analyzed after 12 h of recovery following OGD and normalized with control conditions. All Ct values were normalized relative to the internal control gene, β-actin, using StepOne software v2.3 (Thermo Fisher, USA).

### Immunoblotting and immunofluorescence

Western blots were performed as previously described. 12 h after OGD recovery, cells were homogenized in ice-cold RIPA buffer supplemented with a protease inhibitor mix. Cells were scraped and proteins denatured by incubating at 100 °C for 10 min prior to storage at –80 °C. Protein concentrations were determined using the Dc Protein Assay Kit II before equal amounts of protein were separated under denaturing conditions using 10% Bis-Tris gels. Following transfer onto polyvinylidene fluoride membranes, and blocking in 5% skimmed milk by shaking for 2 h at room temperature, the membranes were incubated at 4 °C overnight in primary antibody prepared in Tris-buffered saline solution containing 0.1% Tween 20 (TBS-Tween). All primary antibodies were diluted 1:1000 (v/v). Membranes were washed and incubated for 1 h at room temperature in a 1:5000 (v/v) dilution of a horseradish peroxidase-conjugated secondary antibody prepared in TBS-Tween. After additional washes, protein bands were visualized using Bio-Rad ChemiDoc XRS (Bio-Rad, USA). Where indicated, expression levels were normalized to β-actin from a parallel western blot. Semi-quantitative analysis of immunoblotting results was performed using ImageJ software (NIH, Bethesda, MD, USA) to determine the mean relative densities of each protein band compared to the control.

For immunofluorescence experiments, cells were treated as described above. Anti- RIPK3 and anti-MLKL primary antibodies (both diluted 1:200) were added separately to the fixed cells and incubated overnight at 4 °C. Incubation with the appropriate fluorescently-labeled secondary antibodies (1:50) for 2 h at RT. Fluorescence images were captured using an Olympus DP71 fluorescence microscope (Olympus, USA).

### Ubiquitination assays

For ubiquitination experiments, cells were treated with a proteasome inhibitor MG132 (10 μM) in the last 6 h before lysis with the IP buffer supplemented with 1 mM PMSF and 10 mM iodoacetamide. Whole-cell lysates obtained by centrifugation were incubated with anti-RIPK3 derived from rabbit or anti-IgG derived from rabbit overnight at 4 °C followed by 4 h incubation with protein G sepharose beads (GE Healthcare). The immunocomplexes were then washed with IP buffer for three times and separated by SDS–PAGE for further western blotting assay. For Western blot analysis anti-RIPK3 derived from mouse were used to test the quantity of RIPK3.

### Flow cytometry

Ratio of PI-positive cells represented the level of necroptosis as previously described. Flow cytometry to measure the ratio of propidium iodide (PI)-positive cells was conducted according to the manufacturer’s instruction with some modifications. After OGD recovery (OGDR) for 12 h, the ratio of PI-positive cells was quantified using an AnnexinV-FITC Apoptosis Detection Kit by Flow Cytometry (MACSQuantTM, Miltenyi Biotech, Bergisch-Gladbach, Germany).

### Analysis and statistics

Results are presented as means ± S.E.M. of the indicated number of independent experiments. Statistical significance was assessed by one-way analysis of variance (ANOVA), followed by Bonferroni’s test using SPSS21.0 software. Semi-quantitative analysis of immunoblotting results was performed to determine the mean relative densities of each protein band compared to control(ImageJ, NIH, Bethesda, MD, USA).

## Electronic supplementary material

supplementary information
