# Author Correction: Anisomycin prevents OGD-induced necroptosis by regulating the E3 ligase CHIP

**DOI:** 10.1038/s41598-021-04253-0

**Published:** 2021-12-28

**Authors:** Mi-bo Tang, Yu-sheng Li, Shao-hua Li, Yuan Cheng, Shuo Zhang, Hai-yang Luo, Cheng-yuan Mao, Zheng-wei Hu, Jonathan C. Schisler, Chang-he Shi, Yu-ming Xu

**Affiliations:** 1 Department of Neurology, The First Affiliated Hospital of Zhengzhou University, Zhengzhou University, Zhengzhou, 450000 Henan China; 2 The Institute of Clinical Medicine, The First Affiliated Hospital of Zhengzhou University, Zhengzhou, China; 3 McAllister Heart Institute, Chapel Hill, NC 27514 USA; 4 Department of Cardiology, The University of North Carolina at Chapel Hill, Chapel Hill, NC 27514 USA

Correction to: *Scientific Reports* 10.1038/s41598-018-24414-y, published online 23 April 2018

This Article contains an error in Figure 1A. As a result of a mistake in figure assembly, the band for p-MLKL is incorrect. The correct Figure 1 and accompanying legend appear below.

**Figure 1:**
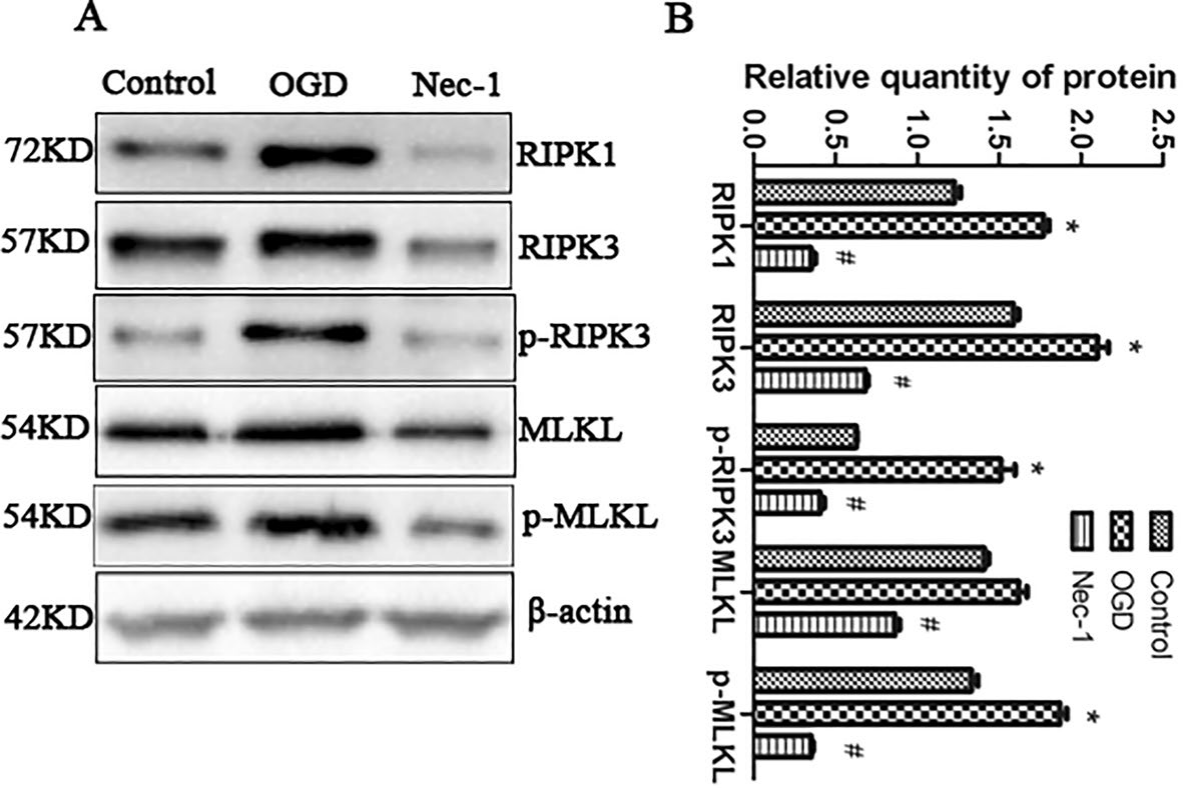
OGD challenge induces necroptotic cell death. (**A–B**) Representative western blot, with β-actin used for normalization. RIPK1, RIPK3, p-RIPK3 and p-MLKL in control cells, cells challenged by OGD which were pre-treated with or without Nec-1 (50 μM) were tested. The bars represent the mean ± SEM of five independent experiments. Significant differences *p < 0.05 vs. Control, ^#^p < 0.05 vs. OGD. Related blots are shown in Supplementary Fig. S1.

This change does not affect the conclusions of the Article.

