# Supplementary material for: Anisomycin prevents OGD-induced necroptosis by regulating the E3 ligase CHIP
Source: Sci Rep. 2018 Apr 23;8:6379. doi: 10.1038/s41598-018-24414-y (PMC5913227; doi:10.1038/s41598-018-24414-y)
Supplement: Supplementary file 1 — supplementary information [file 41598_2018_24414_MOESM1_ESM.doc]

**Anisomycin prevents OGD-induced necroptosis by regulating the** **E3 ligase CHIP**

Mi-bo Tang^1, 2^, Yu-sheng Li^1^, Shao-hua Li^1, 2^, Yuan Cheng^1, 2^, Shuo Zhang^1, 2^, Hai-yang Luo^1, 2^, Cheng-yuan Mao^1^, Zheng-wei Hu^1, 2^, Jonathan C. Schisler^3,4^, Chang-he Shi^1^*, Yu-ming Xu^1^*

^1^: Department of Neurology, The First Affiliated Hospital of Zhengzhou University, Zhengzhou University, Zhengzhou, 450000, Henan, China.

^2^: The Institute of Clinical Medicine (The First Affiliated Hospital of Zhengzhou University, Zhengzhou, China)

^3^：McAllister Heart Institute

^4^：Department of Cardiology, The University of North Carolina at Chapel Hill, Chapel Hill, NC

27514, USA

*: Corresponding Authors: Yuming Xu, Changhe Shi
Address：Department of Neurology, The First Affiliated Hospital of Zhengzhou University, Zhengzhou University. 1 Jian-she east road, Zhengzhou 450000, Henan, China
Tel: +86-371-66862132, Fax: +86-371-66862132

E-mail: xuyuming@zzu.edu.cn, shichanghe@gmail.com

**Supplementary Figure1**

**Figure 1: OGD challenge induces necroptotic cell death**

Images of western blots presented in the main manuscript as figure 1 (A). Irrelevant lanes used for another experiment that were prepared and run on these gels, but not part of this study, have been removed. Our membranes were cut immediately after transfer to incubate in different primary antibodies, which were probed individually for the proteins of interest, as appropriate for their molecular weights. Protein bands were visualized using Bio-Rad ChemiDoc XRS (Bio-Rad, USA) with different time of exposure for each band.

RIPK1


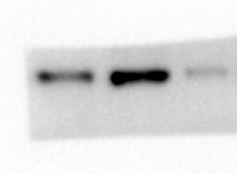


RIPK3


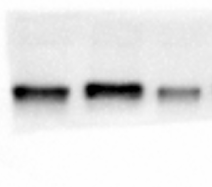


p-RIPK3


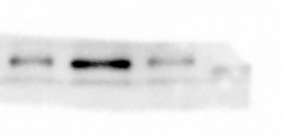


MLKL


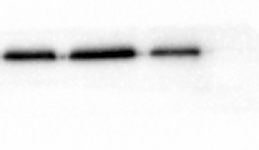


p-MLKL


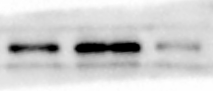


β-actin


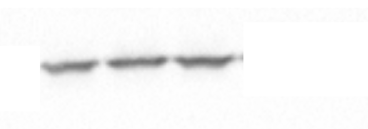


**Supplementary Figure 2**

**Figure 3: Anisomycin functions as an anti-necroptotic factor in OGD-challenged N2a cells by upregulate CHIP**

Images of western blots presented in the main manuscript as figure 3 (A). Irelevant lanes used for another experiment that were prepared and run on these gels, but not part of this study, have been removed. Our membranes were cut immediately after transfer to incubate in different primary antibodies, which were probed individually for the proteins of interest, as appropriate for their molecular weights. Protein bands were visualized using Bio-Rad ChemiDoc XRS (Bio-Rad, USA) with different time of exposure for each band.

**JNK**

**
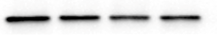
**

**p-JNK**

**
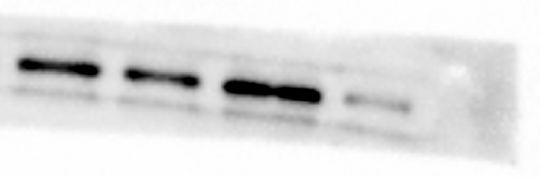
**

**CHIP**

**
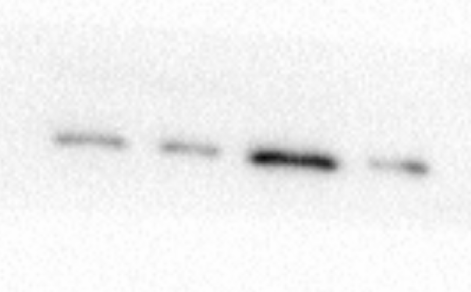
**

**RIPK3**

**
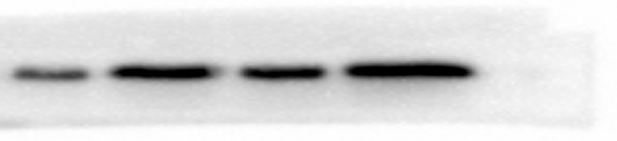
**

**p-MLKL**

**
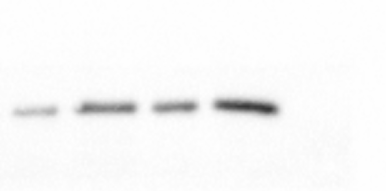
**

**β-actin**

**
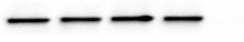
**

**Supplementary Figure3**

**Figure 4: Anisomycin functions as an anti-necroptotic factor in OGD-challenged primary cultured hippocampal neurons and upregulate CHIP**

Images of western blots presented in the main manuscript as figure 4 (A). Irelevant lanes used for another experiment that were prepared and run on these gels, but not part of this study, have been removed. Our membranes were cut immediately after transfer to incubate in different primary antibodies, which were probed individually for the proteins of interest, as appropriate for their molecular weights. Protein bands were visualized using Bio-Rad ChemiDoc XRS (Bio-Rad, USA) with different time of exposure for each band.

JNK


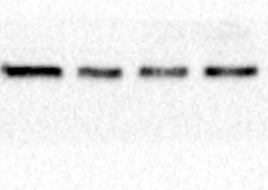


p-JNK


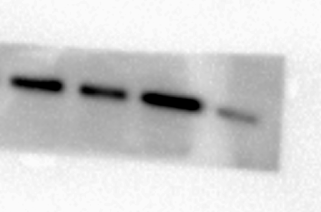


CHIP


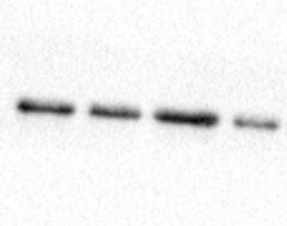


RIPK3


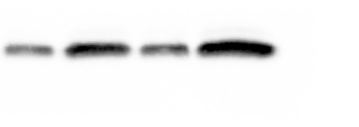


p-MLKL


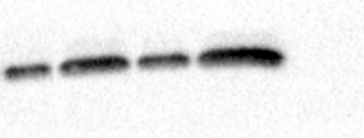


β-actin


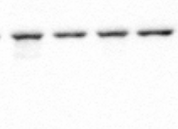


**Supplementary Figure4**

**Fig 5 Anisomycin increase the endogenous RIPK3 ubiquitination level in OGD challenged N2a cells**

Images of ubiquitination presented in the main manuscript as figure 5. Our membranes were cut immediately after transfer to incubate in Ub and RIPK3. Protein bands were visualized using Bio-Rad ChemiDoc XRS (Bio-Rad, USA) with different time of exposure for each band.

Ub


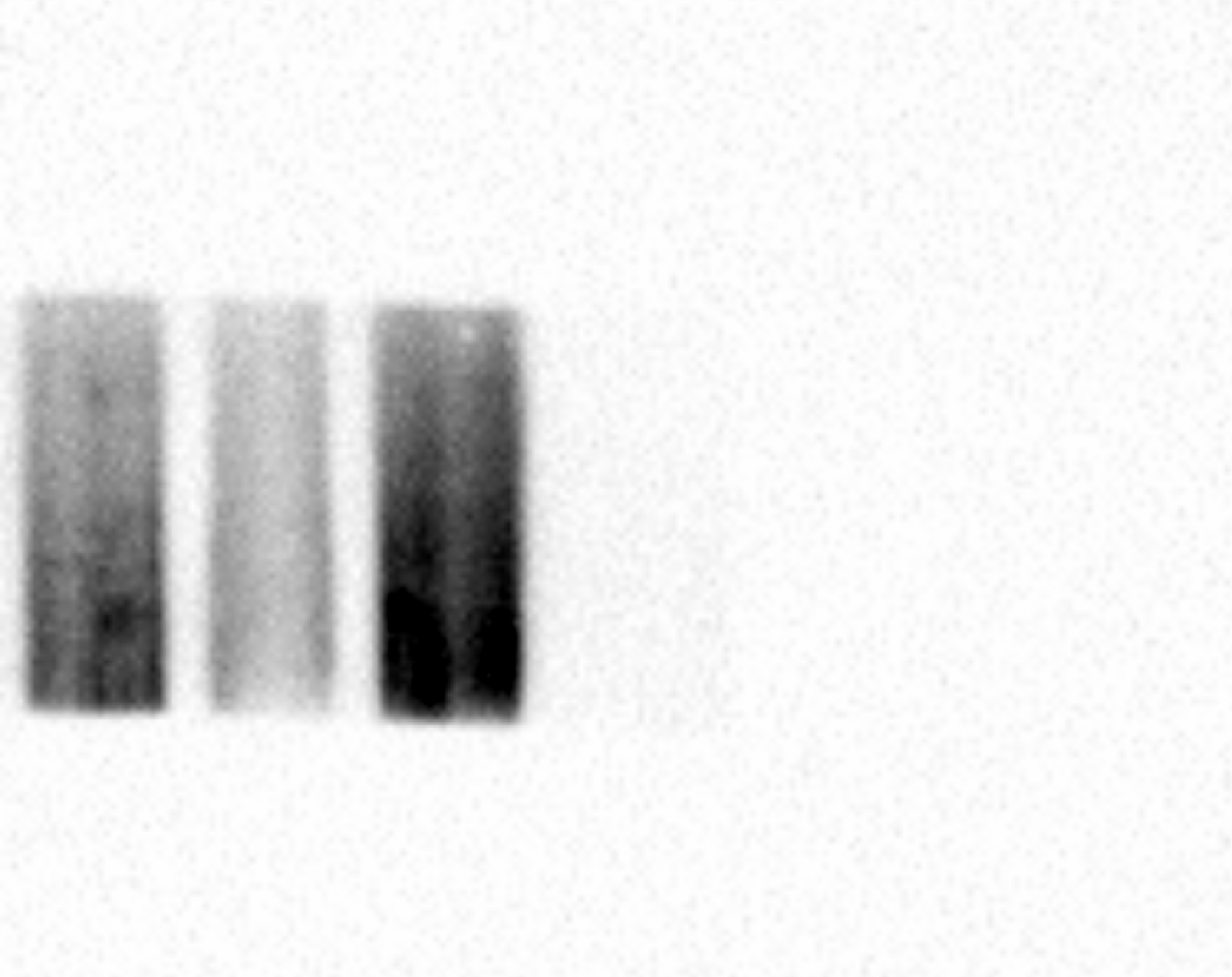


RIPK3


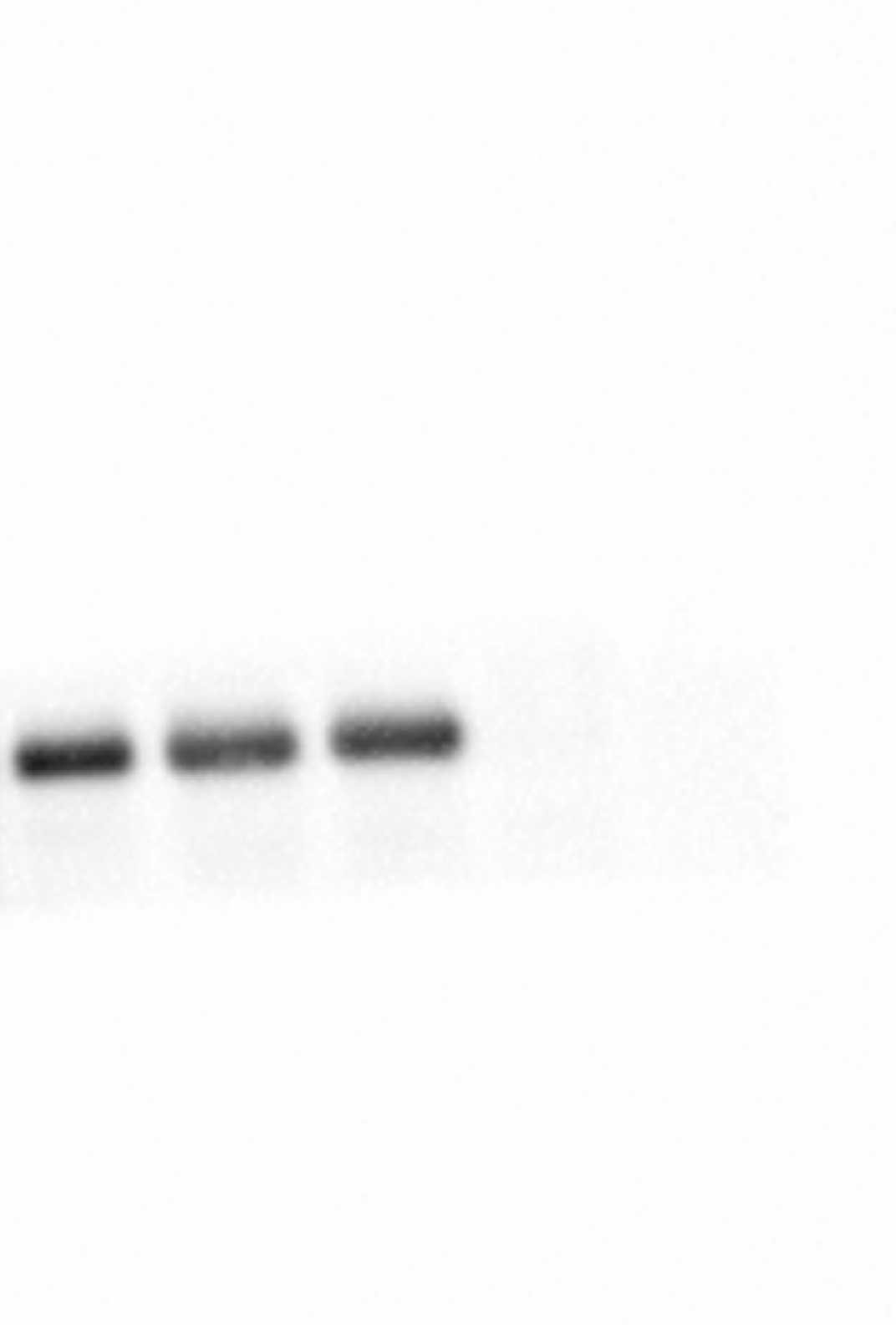


WCE--RIPK3


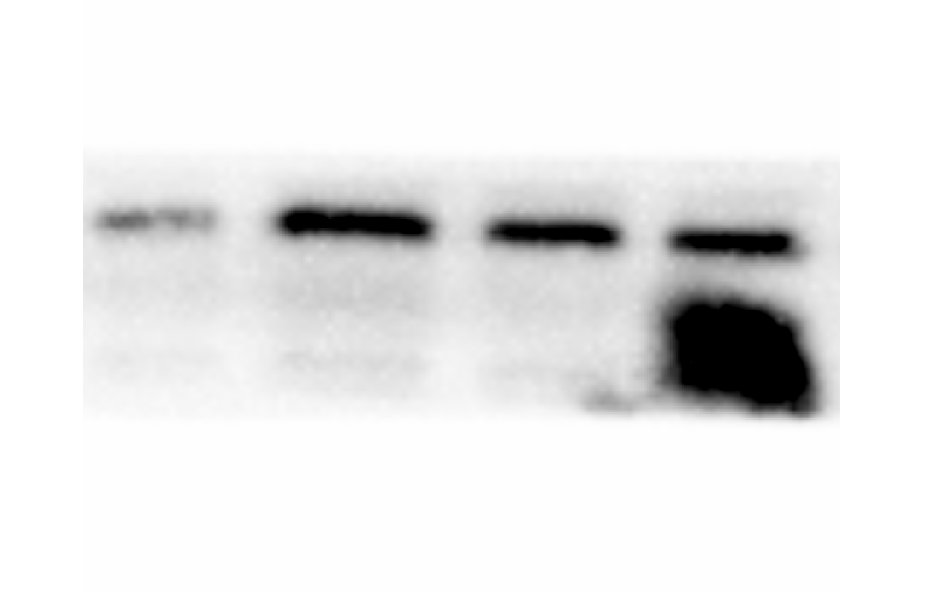


WCE—β-actin


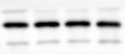


**Supplementary Figure 5**

**Figure6: CHIP functions as an anti-necroptotic factor in OGD-challenged cells**

Images of western blots presented in the main manuscript as figure 6 (D). Irelevant lanes used for another experiment that were prepared and run on these gels, but not part of this study, have been removed. Our membranes were cut immediately after transfer to incubate in different primary antibodies, which were probed individually for the proteins of interest, as appropriate for their molecular weights. Protein bands were visualized using Bio-Rad ChemiDoc XRS (Bio-Rad, USA) with different time of exposure for each band.

**CHIP**

**
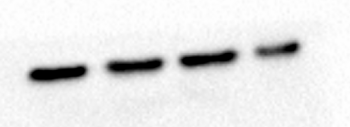
**

**RIPK3**

**
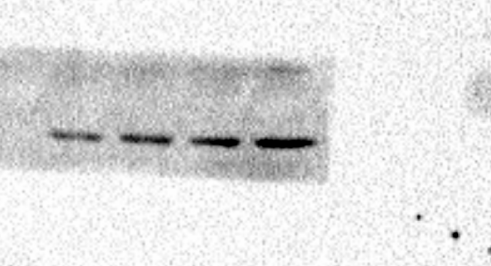
**

**p-MLKL**

**
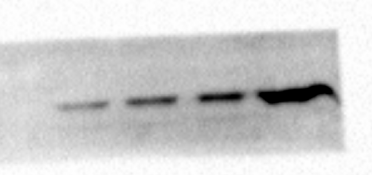
**

**β-actin**

**
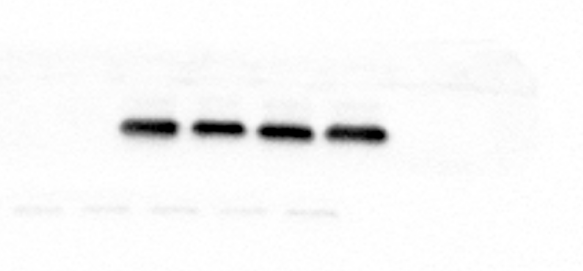
**

**Supplementary Figure 6**

**Figure 7: CHIP is important for reducing RIPK3 levels in anisomycin treated OGD-challenged N2a cells**

Images of western blots presented in the main manuscript as figure 7 (A). Irelevant lanes used for another experiment that were prepared and run on these gels, but not part of this study, have been removed. Our membranes were cut immediately after transfer to incubate in different primary antibodies, which were probed individually for the proteins of interest, as appropriate for their molecular weights. Protein bands were visualized using Bio-Rad ChemiDoc XRS (Bio-Rad, USA) with different time of exposure for each band.

**CHIP**

**
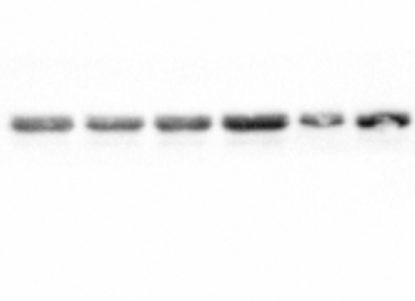
**

**RIPK3**

**
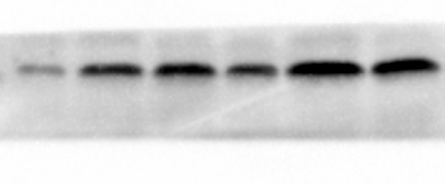
**

**β-actin**

**
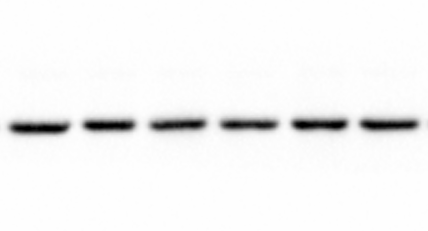
**

**Supplementary Figure 7**

**Figure 8: CHIP functions as an anti-necroptotic factor in a domain-dependent manner**

Images of western blots presented in the main manuscript as figure 8（E）. Irelevant lanes used for another experiment that were prepared and run on these gels, but not part of this study, have been removed. Our membranes were cut immediately after transfer to incubate in different primary antibodies, which were probed individually for the proteins of interest, as appropriate for their molecular weights. Protein bands were visualized using Bio-Rad ChemiDoc XRS (Bio-Rad, USA) with different time of exposure for each band.

**CHIP**

**
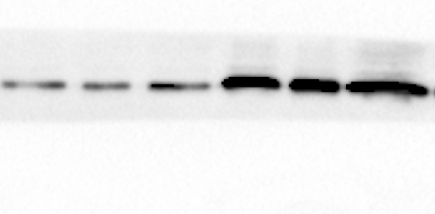
**

**RIPK3**

**
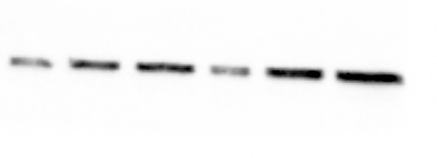
**

**MLKL**

**
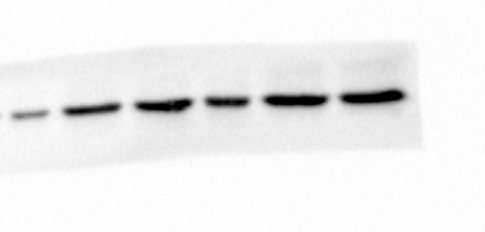
**

**p-MLKL**

**
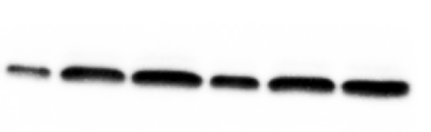
**

**β-actin**

**
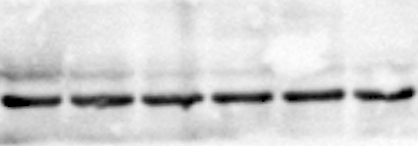
**
